# Non-contrast MRI Radiomics Nomogram for Predicting Recurrence and Progression-free Survival in Cervical Cancer following Concurrent Chemoradiotherapy

**DOI:** 10.2174/0115734056449836260717192214

**Published:** 2026-07-23

**Authors:** Wei Wang, Xiaoting Li, Yueqi Jiang, Yiheng Lu, Min Gao, Yunong Gao, Yun Tian, Jing You, Xiaofan Li, Kun Cao, Hong Zheng

**Affiliations:** 1 Department of Gynecologic Oncology, Key Laboratory of Carcinogenesis and Translational Research (Ministry of Education/Beijing), Peking University Cancer Hospital & Institute, Hai Dian District, Beijing 100142, China; 2 Department of Radiology, Key Laboratory of Carcinogenesis and Translational Research (Ministry of Education/Beijing), Peking University Cancer Hospital & Institute, Hai Dian District, Beijing 100142, China; 3 Graduate Group in Applied Mathematics and Computational Science, University of Pennsylvania, Philadelphia, PA 19104, United States; 4 Department of Psychology, Beijing Normal University, No. 19, Xinjiekouwai St, Haidian District, 100875, Beijing, China; 5 School of Artificial Intelligence, Beijing Normal University, No. 19, Xinjiekouwai St, Haidian District, 100875, Beijing, China; 6 Department of Radiotherapy Oncology, Key Laboratory of Carcinogenesis and Translational Research (Ministry of Education/Beijing), Peking University Cancer Hospital & Institute, Hai Dian District, Beijing 100142, China

**Keywords:** Cervical carcinoma, MRI, Progression-free survival, Radiomics, Concurrent chemoradiotherapy, Kaplan–Meier analysis, MRI

## Abstract

**Background:**

Accurate prognostic stratification following concurrent chemoradiotherapy (CCRT) for cervical cancer remains challenging. Existing approaches often rely on contrast-enhanced magnetic resonance imaging (MRI), which limits applicability in patients with renal dysfunction and in resource-limited settings. We aimed to develop and validate a non-contrast MRI-based prognosis prediction model combined with clinical predictors to estimate recurrence risk and progression-free survival (PFS).

**Materials and Methods:**

A total of 145 cervical cancer patients from two centers were retrospectively analyzed. The model-building cohort (n=114) was used to extract radiomics features from pre-treatment MRI, and an extreme gradient boosting classifier was developed. Clinical predictors were identified through univariate Cox and multivariate logistic regression analyses. A nomogram integrating radiomics and clinical variables was then constructed. External validation was performed in an independent cohort (n=31). Model performance was assessed by discrimination, calibration, and its ability to stratify patients into low- and high-risk groups for predicting 5-year PFS.

**Results:**

The proposed method categorized patients into well-defined risk groups. Survival results varied greatly among groups. Kaplan–Meier analysis indicated that patients with high risk had a worse 5-year PFS compared to those with low risk. This result was observed in both the construction cohort (*P* < 0.001) and the external validation cohort (*P* = 0.012). The prognostic performance was comparable to that of contrast-enhanced MRI–based models. A significant point to note is that no gadolinium administration was necessary.

**Discussion:**

This study develops a radiomics nomogram that relies on non-contrast MRI. The model offers strong and tailored prognostic information. Imaging features and clinical predictors combined, which improved predictive accuracy. The approach increases accessibility, particularly for patients contraindicated for contrast or in low-resource settings.

**Conclusion:**

We developed and validated a non-contrast MRI radiomics nomogram that accurately predicts recurrence and 5-year PFS following CCRT in cervical cancer. The model offers a clinically applicable, cost-effective tool for personalized risk stratification.

## 1 INTRODUCTION

Cervical cancer is a major cause of cancer-related morbidity and mortality in women worldwide. Low- and middle-income countries bear a particularly heavy burden. According to the Global Cancer Observatory's 20 estimates, there were 604,127 new cases and 341,831 deaths globally. Countries with lower Human Development Indices (HDI) experience a significantly higher disease burden [1]. Concurrent chemoradiotherapy (CCRT) is the standard treatment for patients with locally advanced and postmenopausal cervical cancer, as recommended by the National Comprehensive Cancer Network (NCCN) guidelines [2]. However, roughly 30–40% of patients develop disease recurrence or distant metastasis after definitive CCRT [3]. These events are linked to limited therapeutic options and poor survival outcomes [4]. Therefore, accurate prognostic stratification after CCRT is crucial. Reliable predictors of recurrence and disease-free survival (DFS) are useful for identifying high-risk patients. Such patients might get better treatment strategies and more follow-up. This approach might enhance long-term survival outcomes.

Radiomics has emerged as a powerful, non-invasive method for characterizing tumor biology. It enables the high-throughput extraction of quantitative features from medical images. These features include information about disease development. So, radiomics can enhance diagnostic, prognostic, and predictive performance [5-8]. By converting routine medical images into high-dimensional, mineable data, radiomics offers a quantitative framework for analysis [9]. This framework can be incorporated into clinical decision support systems to support clinical decision-making. A lot of quantitative features can be extracted from CT, MRI, and PET images *via* high-throughput computing techniques [10-12]. While imaging is crucial, it is not the sole determinant of clinical decisions. Radiomics features (*e.g*., intensity, shape, texture, wavelet features) provide insights into tumor phenotype and the tumor microenvironment, complementing data from other sources such as clinical features, therapeutic response, or genomics [13]. Integrating radiomics with other data types and correlating them with outcomes enables the development of accurate and reliable CDSSs for outcome prediction [14].

However, most existing radiomics studies on cervical cancer have relied on contrast-enhanced MRI or CT [15-19]. Although some studies have explored diffusion-weighted imaging (DWI) for prognosis, such as histogram analysis of apparent diffusion coefficients (ADC) [20], there remains a need for models based solely on routinely available non-contrast sequences. Non-contrast MRI sequences such as T2-weighted imaging (T2WI) and high b-value DWI are more widely available and avoid the risks and costs associated with gadolinium contrast [1]. Therefore, we developed a non-contrast MRI–based prognosis prediction model that integrates features extracted from T2WI and DWI with key clinical predictors. Our goal was to establish a clinically accessible and accurate tool for predicting recurrence and 5-year progression-free survival (PFS) in cervical cancer patients following CCRT.

## 2 MATERIALS AND METHODS

### 2.1 Study Population

This retrospective study was approved by the Ethics Review Committee of Beijing Cancer Hospital, China (reference number 2021KT49), with informed consent waived. Two patient cohorts were retrospectively identified and extracted from the institutional electronic medical record (EMR) system. The model construction cohort (Group A, n=114) consisted of cervical cancer patients treated with definitive CCRT at the main campus of Peking University Cancer Hospital between January 2012 and December 2013. The external validation cohort (Group B, n=31) included patients treated between January and June 2014 at Beijing Cancer Hospital Southern Branch, ensuring both temporal and geographic separation for model testing. Clinical staging was reevaluated jointly by a radiologist and a gynecologic oncologist based on the original clinical data and the 2018 International Federation of Gynecology and Obstetrics (FIGO) staging criteria.

Inclusion criteria were: (a) FIGO stage IB-IVA disease, (b) histology of squamous cell carcinoma or adenocarcinoma, (c) definitive CCRT completed, (d) pretreatment pelvic MRI performed within 30 days before treatment initiation, and (e) available follow-up data. Clinical data included age, histological differentiation, serum squamous cell carcinoma antigen (SCC-Ag) level, lymph node status on MRI, discharge date, and last follow-up. All patients who met the above inclusion criteria were included in this study. No additional exclusion criteria were applied. The study flowchart is shown in Fig. (**1**).

A total of 145 patients were analyzed, including 114 in the construction cohort and 31 in the external validation cohort. All enrolled patients were female, this in line with the epidemiology of cervical cancer. In the construction cohort, the mean age was 54.16 ± 5.70 years. In the validation cohort, the mean age was 50.96 years with a standard deviation of 9.39 years. The age difference between cohorts was not statistically significant (*P* = 0.097). The baseline characteristics between the two cohorts were comparable, as indicated in Table **1**. The distributions of FIGO stage, histological type, tumor differentiation, and progression rates showed no significant differences (all *P* > 0.05). These results show that the validation cohort is representative of the model development cohort.

There were a total of 58 progression events observed across both cohorts. This number meets the requirement of having at least 10 outcome events per predictor variable for logistic regression analysis. All eligible patients were included in the study period. This was enough to guarantee model stability and reliable external validation.

### 2.2 Treatment and Follow-up

Patients received definitive concurrent radiotherapy and chemotherapy. External beam radiotherapy (EBRT) was conducted with 45 Gy in 25 fractions. Nodal boost was conducted at 60 Gy in 25 fractions for the involved lymph nodes. Intracavitary brachytherapy (ICBT) (28 Gy in 4 fractions) was delivered with standard applicators. Cisplatin was given concurrently during radiotherapy at a dosage of 32~40 mg/m^2^ weekly.

Follow-up consisted of examinations every three months for two years, every six months in 3~4 years, and annually thereafter. PFS was defined as the interval between completion of CCRT and documented disease progression (local recurrence, distant metastasis, or cancer-specific death) or last follow-up.

### 2.3 MRI Acquisition

All examinations were performed on 1.5-T MR scanners (GE optima 1.5 T MR360 and GE signa 1.5 T EchoSpeed Plus) with four-channel or eight-channel torso phased array body coils. The scanning range was from the level of the anterior superior iliac spine to the inferior level of the symphysis pubis. All patients were required to fast for at least 2 h before the examination. The MR sequences included T1WI, T2WI, and two b-value DWI. The standard MR scan protocol was identical each time and was as follows: (a) axial T1WI: fast spin-echo (FSE) sequence, repetition time (TR) = 726 ms, echo time (TE) = 12 ms, matrix size = 288 × 256, field of view (FOV) = 26–36 cm, slice thickness/intersection gap = 8/1 mm, number of excitations (NEX) = 1; (b) axial T2WI: FSE-XL, TR = 5182 ms, TE = 80 ms, matrix size = 352 × 320, FOV = 26~30 cm, slice thickness/intersection gap =8/1 mm, NEX = 2; (c) sagittal T2WI: FSE-XL, TR = 4138 ms, TE = 102 ms, matrix size = 320 × 288, FOV = 24~36 cm, slice thickness/intersection gap = 5/1 mm, NEX = 4; (d) axial DWI: single-shot echo-planar imaging sequence, TR = 3500 ms, TE = minimum time, matrix size = 128 × 128, FOV = 36 cm, slice thickness/intersection gap = 8/1 mm, NEX = 4, b-value = 0 and 1000 s/mm^2^.

### 2.4 Image Segmentation and Rradiomics Feature Extraction

Tumor regions of interest (ROIs) were manually delineated on T2WI for all patients by two experienced radiologists (with 10 and 12 years of experience in MR gynecologic imaging, respectively). Following initial independent segmentation, the Dice similarity coefficient (DSC) was calculated to assess inter-observer agreement. For cases with inconsistent overlap or ambiguous boundaries, the two radiologists conducted a joint review to reach a final consensus through discussion. Radiomics features, including first-order, shape, and texture descriptors, were extracted using PyRadiomics, an open-source Python package. Feature selection was performed using the least absolute shrinkage and selection operator (LASSO). A flowchart detailing the tumor segmentation and feature extraction processes is shown in Fig. (**2A** and **B**).

### 2.5 Feature Selection and Model Construction

Feature selection employed LASSO to identify a parsimonious radiomics signature. We compared four classifiers (XGBoost, Support Vector Machine (SVM), Random Forest (RF), and Fully Connected Neural Network (FCNN)) using nested cross-validation within the construction cohort. Model performance was assessed by area under the receiver operating characteristic curve (AUC), accuracy, sensitivity, and specificity. XGBoost demonstrated the highest predictive performance and was used in subsequent prognostic modeling. Clinical candidate predictors (age, histological differentiation, pretreatment SCC-Ag, MRI lymph node status) were evaluated by univariate analysis and then multivariable Cox proportional hazards regression. A combined model incorporated the radiomics signature and independent clinical predictors. The combined model was used to construct a nomogram for predicting recurrence and 5-year PFS. Patients were stratified into high- and low-risk groups using the optimal cutoff determined by the Youden index. Figure **2C** and **D** illustrate the detailed process of key feature selection and model construction.

### 2.6 Statistical Analysis

Continuous variables were expressed as means and standard deviations, while categorical variables were represented by absolute numbers and proportions. The comparison of features between the model construction cohort and the testing cohort was performed using Student's t-test and the chi-square test. A combined model integrating clinical and radiomic features was built using logistic regression, and a nomogram predicting 5-year PFS was developed. The optimal cutoff for stratifying patients was determined using the maximum Youden index. Survival analysis was conducted using Kaplan-Meier curves with log-rank tests. Statistical analyses were performed using R statistical software (version 4.4.2), with a two-sided *P*-value <0.05 considered statistically significant.

## 3 RESULTS

### 3.1 Patient Characteristics

Patient characteristics are summarized and compared in Table **1**. The testing cohort had a statistically significantly higher proportion of patients with positive lymph nodes and elevated SCC-Ag (*P*=0.007 and *P* =0.016, respectively). Other characteristics were comparable between the two cohorts (*P*>0.05). Within the model construction cohort, the training set included 93 patients and the validation set included 21 patients. Disease progression occurred in 44 patients in the model construction cohort (36 in training, 8 in validation) and 14 patients in the testing cohort. Median PFS was 63 months (range: 3~155 months) in the model construction cohort and 50 months (range: 5~126 months) in the testing cohort.

### 3.2 Radiomics Features and Model Comparison

After filtering and LASSO selection, features with non-zero coefficients at the optimal λ value (λ = 0.05) were retained, yielding 19 key features (3 first-order, 5 shape, 11 texture features). XGBoost achieved superior discrimination compared with SVM, RF, and FCNN (training AUC 0.989, internal validation AUC 0.952, external AUC 0.832). Detailed sensitivity, specificity, and accuracy metrics are provided in Table **2**.

### 3.3 Clinical Predictors and Combined Model

A clinical model was first developed using logistic regression. Histological differentiation (OR = 3.58, 95% CI: 1.06~12.14) and lymph node metastasis (OR = 70.31, 95% CI: 5.26~598.20) were identified as independent predictors of recurrence. The clinical model achieved a balanced diagnostic performance, with sensitivity of 0.629 in the training set, 0.778 in the validation set, and 0.857 in the test set, respectively. The radiomics model showed good diagnostic ability with sensitivity and specificity of 0.857 and 0.882 in the test set. When radiomics features were integrated with clinical variables, the combined model further improved overall prediction. It achieved sensitivity and specificity of and 0.857 and 0.882 in the test set. Importantly, the combined model significantly outperformed the radiomics model alone (*P*=0.017). A nomogram based on this model was developed to predict 5-year PFS individually, as illustrated in Fig. (**3**). The nomogram offers a practical method for making personalized clinical decisions.

### 3.4 Survival Stratification

Figure **4** presents the calibration curve of the combined model in the validation cohort. The predicted probabilities matched the observed results well. Bootstrap-corrected calibration with 1000 resamples indicated stable accuracy, with a mean absolute error of 0.024. In the external test cohort, calibration remained satisfactory, with a mean absolute error of 0.099. Decision curve analysis further confirmed the clinical utility of the model. In the validation cohort, the combined model provided a consistent net benefit across risk thresholds ranging from approximately 10% to 85%, outperforming both “treat-all” and “treat-none” strategies. Similarly, in the test cohort, the model yielded positive net benefit across a clinically meaningful range of threshold probabilities (approximately 15~80%).

Based on the model-defined cutoff, 63 patients were classified as low-risk and 51 as high-risk in the construction cohort. In the independent test cohort, 24 patients were categorized as low-risk and 7 as high-risk. Kaplan-Meier survival analyses with log-rank testing demonstrated significantly inferior PFS in the high-risk group compared with the low-risk group, both in the construction cohort (*P* < 0.001) and the test cohort (*P* = 0.012), as shown in Fig. (**5**).

## 4 DISCUSSION

This study presents a novel non-contrast MRI radiomics nomogram that integrates T2WI- and DWI-derived features with clinical predictors to predict recurrence and PFS in cervical cancer patients following CCRT. The model has a strong prognostic performance. Its effectiveness was validated independently in a validation cohort. The model successfully categorized patients into well-defined risk groups. Survival outcomes varied a lot between these.

Our approach is not based on contrast-enhanced MRI or CT, but it uses widely available non-contrast MRI sequences. This design doesn't require gadolinium administration. It enhances accessibility and lowers the cost of examinations. It also expands the applicability to patients with renal impairment and resource-limited settings [1]. The proposed nomogram has a prognostic accuracy similar to contrast-enhanced models, which supports its clinical relevance.

From a clinical standpoint, the nomogram enhances the accuracy of prognostic stratification. It accurately detects patients at the highest risk of recurrence. These patients might need to undergo more surveillance or adopt additional treatment strategies. Compared to patients who are classified as low risk, they might have less intensive follow-up. This method could alleviate both psychological stress and financial strain.

Radiomics features and clinical predictors are integrated, which further improves model performance. Lymph node metastasis (OR = 70.31) and histological differentiation (OR = 3.58) were strong prognostic factors. Specificity rose to 8.2%, which is higher than the 76.5% of the radiomics-only model. Clinical interpretability was also enhanced. Kaplan–Meier analysis showed that the combined model has prognostic value. Significant differences in PFS were observed in both the construction cohort (*P* < 0.001) and the external validation cohort (*P* = 0.012).

The radiomics features in the model capture essential tumor biology aspects. Texture features like GLCM cluster prominence and GLSZM gray-level non-uniformity describe intratumoral heterogeneity and spatial complexity [21-23]. Shape features such as surface volume ratio are linked to invasive potential [17, 24]. This non-invasive method enhances the FIGO staging and serum biomarker analysis, including the SCC-Ag. It contributes to the resolution of current prognostic system limitations.

## 5 LIMITATIONS

Though there are these strengths, a few limitations should be acknowledged. First, this was a retrospective study with a relatively small sample size. These factors might restrict the generalizability of the results. Prospective, multicenter validation with larger cohorts is warranted to confirm clinical utility. Second, only MRI-based radiomics features were analyzed; integration with PET/CT or CT radiomics could provide complementary biological information. Third, the follow-up period was relatively short, precluding robust assessment of long-term outcomes. Future work will focus on prospective validation across diverse populations, comparison of XGBoost with emerging deep learning architectures, and assessment of the cost-effectiveness of non-contrast MRI-based strategies in low-resource settings. Future work will focus on prospective validation across diverse populations, comparison of XGBoost with emerging deep learning architectures, and assessment of the cost-effectiveness of non-contrast MRI-based strategies in low-resource settings.

## CONCLUSION

This study demonstrates that a non-contrast MRI radiomics nomogram combining pre-treatment imaging features and clinical predictors accurately predicts recurrence and progression-free survival following CCRT in cervical cancer. This approach provides a reliable tool for risk stratification without requiring gadolinium contrast, making personalized prognostic assessment feasible for patients with renal dysfunction or in resource-limited settings.

## Figures and Tables

**Fig. (1):**
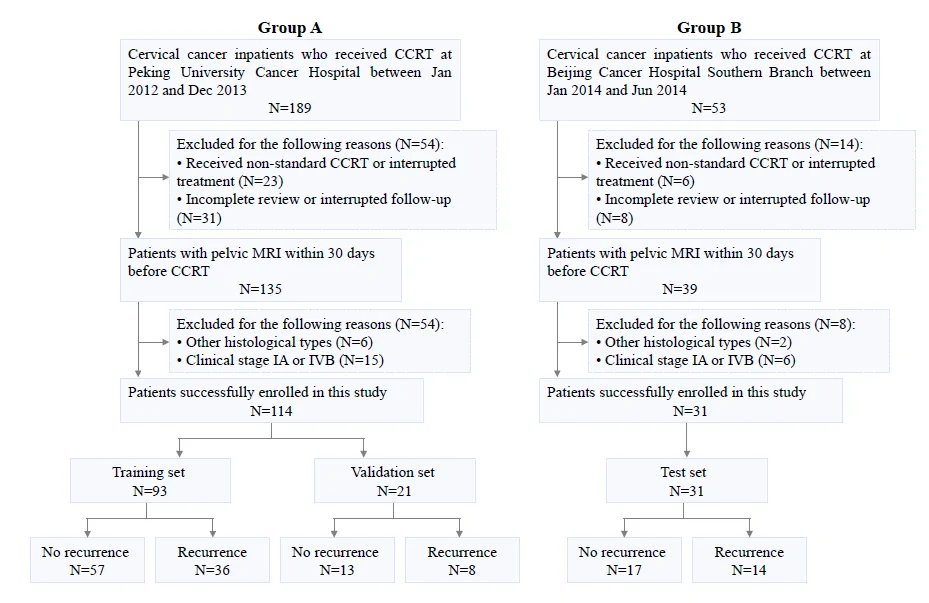
Flowchart of study population with inclusion and exclusion criteria.

**Fig. (2):**
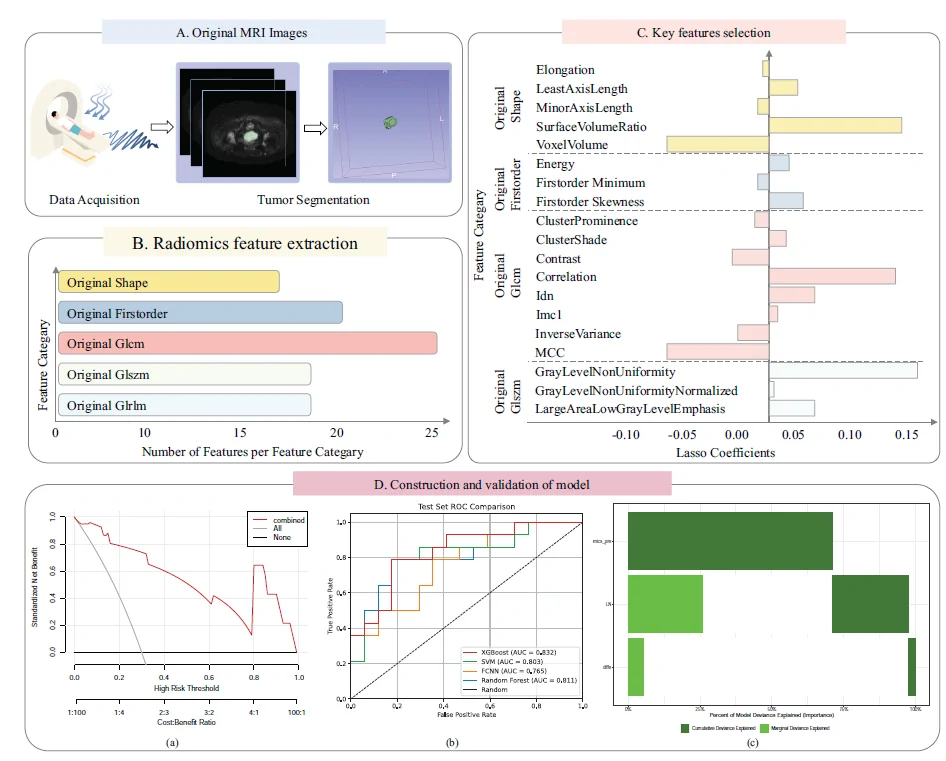
Workflow of MRI Radiomics-based predictive model construction and validation. **A.** Original MRI images and tumor segmentation. **B**. Radiomics feature extraction. **C.** Key feature selection *via* LASSO regression. **D**. Construction and validation of the radiomics, clinical, and combined models with ROC curves, and the importance of clinical variables.

**Fig. (3):**
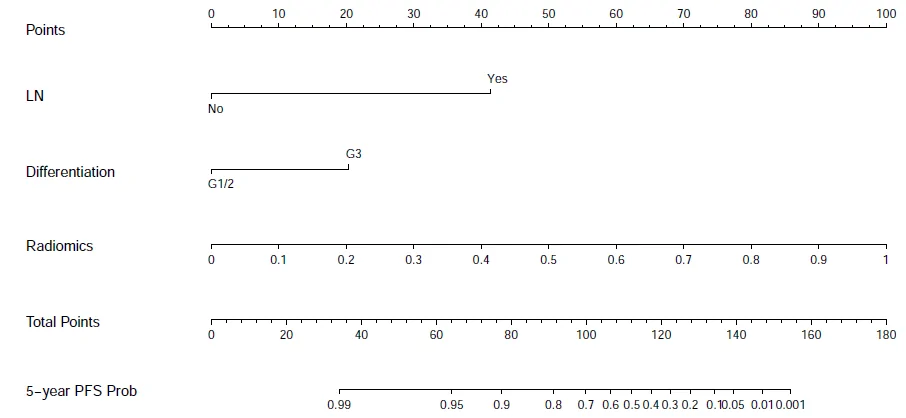
The nomogram of the combined model for predicting 5-year progression free survival.

**Fig. (4A-D):**
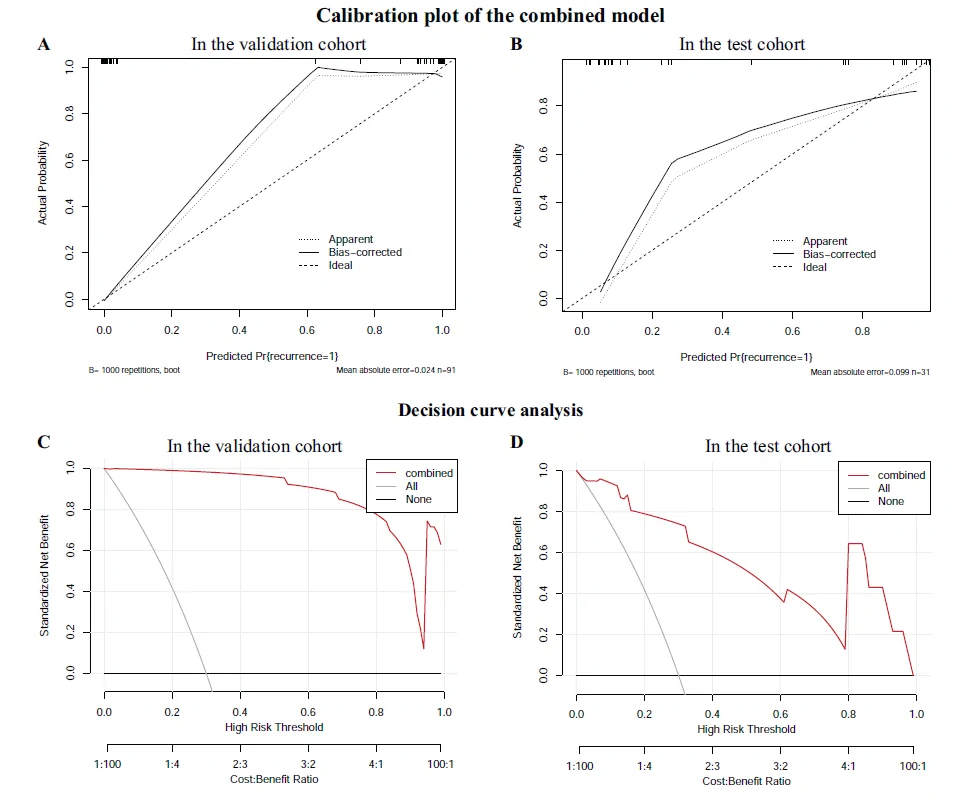
Calibration plot of the combined model and decision curve analysis in the validation and test cohort.

**Fig. (5):**
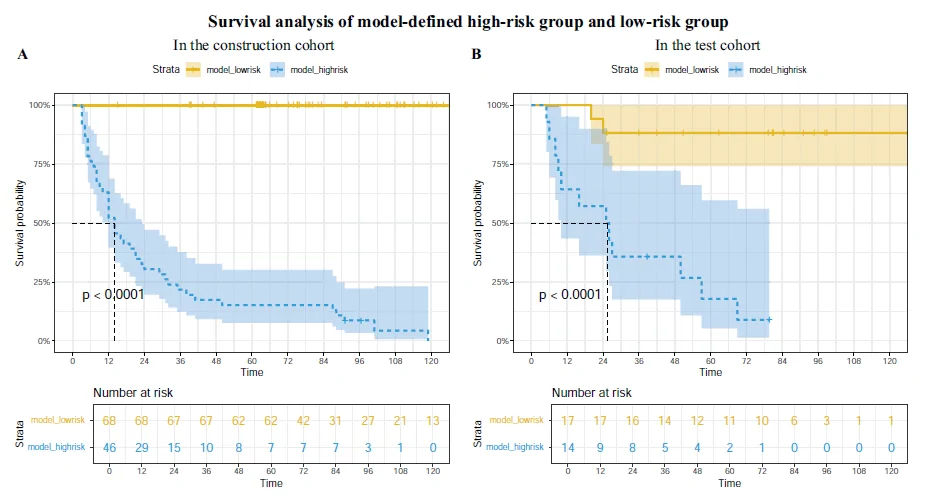
Survival analysis of model-defined high-risk group and low-risk group in the model of construction cohort and test cohort: **A**. Survival analysis in the model of construction cohort, **B**. Survival analysis in the model of test cohort.

**Table 1:** Baseline characteristics of patients in the construction cohort and testing cohort.

**Characteristic**	**Construction Cohort** **(n=114)**	**Testing Cohort** **(n=31)**	*P*
**Age**	54.16±5.70	50.96±9.39	0.097
**Gender**	Female	Female	-
**FIGO stage**			0.648
IB&II III-IVA	89 (78.10%) 25 (21.90%)	23 (74.20%) 8 (25.80%)	
**Histology**			>0.999
Squamous carcinoma	107 (93.90%)	30 (96.80%)	
Adenocarcinoma	7 (6.10%)	1 (3.20%)	
**SCC-Ag(0-1.5ng/ml)**			0.016
High Normal	84 (73.70%) 30 (26.30%)	29 (93.50%) 2 (6.50%)	
**Differentiation**			0.964
G1& G2 G3	52 (45.60%) 62 (54.40%)	14 (45.20%) 17 (54.80%)	
**Lymph node metastasis** Yes	24 (21.10%)	14 (45.20%)	0.007
No	90 (78.90%)	17 (54.80%)	
**Progression** Yes	44 (38.60%)	14 (45.20%)	0.508
No	70 (61.40%)	17 (54.80%)	

**Table 2:** Comparison of parameters for various algorithms model.

**Model**	**XGBoost**	**SVM**	**RF**	**FCNN**
Training set(n=93)				
AUC	0.989 (0.962,1.000)	0.710 (0.597,0.818)	0.984 (0.949,1.000)	0.899 (0.837,0.959)
Cutoff	0.710	0.396	0.480	0.361
Sen	1.000	0.686	1.000	0.943
Spe	0.982	0.714	0.982	0.750
Acu	0.989	0.703	0.989	0.824
Validation set(n=21)				
AUC	0.952 (0.838,1.000)	0.738 (0.492,0.950)	0.984 (0.929,1.000)	0.873 (0.714,1.000)
Sen	1.000	0.778	1.000	1.000
Spe	0.929	0.714	0.929	0.643
Acu	0.957	0.739	0.957	0.783
Test set(n=31)				
AUC	0.832(0.676,0.955)	0.803(0.604,0.946)	0.811(0.654,0.952)	0.765(0.585,0.913)
Sen	0.786	1.000	0.786	1.000
Spe	0.647	0.235	0.765	0.294
Acu	0.710	0.581	0.774	0.613

## Data Availability

All source code is publicly available on Github (https://github.com/Luyiheng-19/MRI-and-clinical-parameters--Based-Radiomics-Cervical-Cancer-Predictionon). Further information is available from the corresponding authors [K.C] and [H.Z] upon request.

## LIST OF ABBREVIATIONS

Acu: Accuracy
AI: Artificial intelligence
ANN: Artificial neural network
AUC: Area under the curve
CCRT: Concurrent chemoradiotherapy
CDSS: Clinical Decision Support System
CI: Confidence interval
C-index: Concordance index
CPF: Clinical prognostic factor
CT: Computed tomography
DFS: Disease-free survival
EBRT: External beam radiotherapy
FCNN: Fully connected neural network
FIGO: International Federation of Gynecology and Obstetrics
G1: High differentiation
G2: Moderate differentiation
G3: Low differentiation
GLCM: Gray-level cooccurrence matrix
GLSZM: Gray-level size zone matrix
HDI: Human Development Index
ICBT: Intracavitary brachytherapy
LVSI: Lymphovascular space invasion
MRI: Magnetic resonance imaging
NCCN: National Comprehensive Cancer Network
OR: Odds ratio
OS: Overall survival
PET: Positron emission tomography
PFS: Progression-free survival
RF: Random Forest
ROC: Receiver operating characteristic
ROI: Region of interest
SCC-Ag: Squamous cell carcinoma antigen
Sen: Sensitivity
Spe: Specificity
SVM: Support Vector Machine
XGBoost: Extreme gradient boosting

## STANDARDS OF REPORTING

STROBE guidelines were followed.
